# Platelet δ-Storage Pool Disease: An Update

**DOI:** 10.3390/jcm9082508

**Published:** 2020-08-04

**Authors:** Arnaud Dupuis, Jean-Claude Bordet, Anita Eckly, Christian Gachet

**Affiliations:** 1 INSERM, EFS Grand Est, BPPS UMR-S 1255, FMTS, Université de Strasbourg, F-67000 Strasbourg, France; Anita.Michel@efs.sante.fr (A.E.); christian.gachet@efs.sante.fr (C.G.); 2 Laboratoire D’hématologie, Hospices Civils de Lyon, 59 Bd Pinel, CEDEX, 69677 Bron, France; jean-claude.bordet@chu-lyon.fr

**Keywords:** blood platelets, storage pool disorder, electron microscopy, inherited platelet disorders

## Abstract

Platelet dense-granules are small organelles specific to the platelet lineage that contain small molecules (calcium, adenyl nucleotides, serotonin) and are essential for the activation of blood platelets prior to their aggregation in the event of a vascular injury. Delta-storage pool diseases (δ-SPDs) are platelet pathologies leading to hemorrhagic syndromes of variable severity and related to a qualitative (content) or quantitative (numerical) deficiency in dense-granules. These pathologies appear in a syndromic or non-syndromic form. The syndromic forms (Chediak–Higashi disease, Hermansky–Pudlak syndromes), whose causative genes are known, associate immune deficiencies and/or oculocutaneous albinism with a platelet function disorder (PFD). The non-syndromic forms correspond to an isolated PFD, but the genes responsible for the pathology are not yet known. The diagnosis of these pathologies is complex and poorly standardized. It is based on orientation tests performed by light transmission aggregometry or flow cytometry, which are supplemented by complementary tests based on the quantification of platelet dense-granules by electron microscopy using the whole platelet mount technique and the direct determination of granule contents (ADP/ATP and serotonin). The objective of this review is to present the state of our knowledge concerning platelet dense-granules and the tools available for the diagnosis of different forms of δ-SPD.

## 1. Introduction

Platelets are small anucleated blood cells whose main physiological role is the formation of a platelet clump to stop bleeding in the case of vascular injury. Blood platelets, which measure 2 to 4 µm in diameter, have multiple organelles in their cytoplasm, two of which are specific to these cells: dense-granules (δ-granules) and alpha granules (α-granules). The contents of these granules are secreted during the activation of platelets, leading to their aggregation [1]. Alpha granules release into the circulation more than 300 different proteins with various activities (stimulation of coagulation, angiogenesis, and inflammation), while dense-granules release mainly small molecules: serotonin, adenyl nucleotides (ADP (adenosine diphosphate) and ATP (adenosine triphosphate)), polyphosphates, and calcium [2]. Released calcium contributes to the coagulation cascade, while secreted ADP and serotonin act in an autocrine and paracrine manner on specific receptors located on the membrane of adjacent platelets to induce their aggregation. In particular, ADP binds to its two G-protein-coupled receptors, P2Y_1_ and P2Y_12_, leading to a change in platelet shape and to amplification of the platelet activation induced by all other agonists including serotonin, thromboxane A2, collagen, immune complexes, and thrombin [3]. As a consequence, a defect in dense-granules or their content decreases platelet activation by reducing the quantities of secreted ADP and serotonin, resulting in a type of platelet function disorder (PFD) called δ-storage pool disease (δ-SPD) [4]. These platelet pathologies are characterized by a heterogeneous hemorrhagic phenotype and are observed in syndromic forms associated with oculocutaneous depigmentation and/or immune deficiencies (Chediak–Higashi disease, Hermansky–Pudlak syndromes), but also in non-syndromic forms, which only affect the platelet lineage. In all cases, the diagnosis of δ-SPD remains complex. It relies on specialized biological tests requiring powerful tools such as electron microscopy to count dense-granules and specific biochemical tests to determine their content. In this review, we propose to summarize the current state of knowledge concerning the origin and function of dense-granules, the different dense-granule deficiencies observed in man and the tools currently available in laboratories for the diagnosis these platelet diseases.

## 2. Platelet Dense-Granules: Origin, Structure, and Function

In contrast to α-granules, whose origin is essentially related to the Golgi apparatus of megakaryocytes, dense-granules are organelles derived from lysosomes [5]. Studies carried out in patients with Hermansky–Pudlak syndrome have revealed that the protein complexes BLOC (biogenesis of lysosome-related organelle complexes) and AP-3 (adaptor protein 3), involved in the biogenesis of these lysosome-derived organelles, are impaired. Three BLOC complexes were identified: BLOC-1, BLOC-2, and BLOC-3. The BLOC-1 complex is composed of at least eight proteins: cappuccino; dysbindin; snapin; BLOC-1 subunit 1, 2, 3, and 5; and pallidin [6]. Pallidin is known to interact with syntaxin 13 [7], a t-SNARE (soluble N-ethylmaleimide-sensitive factor attachment protein receptor) essential for membrane fusion during exocytosis. The BLOC-2 complex consists of three proteins (HPS3, 5, and 6) and is involved in the transport and maturation of the organelles in cooperation with the GTPases RAB32 and RAB38 [8]. The BLOC-3 complex is composed of two proteins [9] and would allow switching of the MRP4 nucleotide transporter to the dense-granule membrane [10]. In addition, the work of Ambrosio et al. [11] in a megakaryocyte lineage (MEG-01) would suggest that the AP-3 complex participates in the orientation of structures derived from late endosomes and expressing the VMAT2 and MRP4 transporters towards a tubular compartment dedicated to the maturation of dense-granules. Studies performed by this team have also confirmed the role of RAB32 and RAB38 GTPases in dense-granule maturation [12,13]. The mechanisms of uptake of highly concentrated small molecules into dense-granules remain only partially identified. Circulating serotonin, produced by entero-chromaffin cells in the gut, is rapidly captured by blood platelets through the membrane receptor SERT and then incorporated into platelet dense-granules via the VMAT2 transporter [14]. Conversely, the mechanism controlling the concentration of calcium in dense-granules is not known. Concerning adenyl nucleotides, the MRP4 transporter is present on the membrane of dense-granules and is thought to participate in the transport of nucleotides from the cytoplasm towards the interior of these organelles [10]. Studies performed by Decouture et al. [15] in MRP4-deficient mice have shown that the MRP4 transporter is largely involved in the sequestration of cAMP in dense-granules during platelet activation.

Mature dense-granules are about 150 nm in diameter and are termed “dense” because their calcium-rich centers are opaque to electrons in transmission electron microscopy. A platelet has between three and eight dense-granules, which contain small molecules: calcium, present at very high concentrations (about 2 M); 90% of the circulating serotonin in the body (400 to 600 ng/10^9^ platelets); and large quantities of nucleotides (ATP and ADP at concentrations of 0.6 and 0.4 M, respectively). The adenyl nucleotides stored in the dense-granules (non-metabolic pool) represent 60% of the amount contained in a whole platelet. The remaining 40% (metabolic pool) is present in free form in the cytoplasm or bound to actin in the mitochondria. These two pools are not interchangeable. Platelets contain predominantly ATP distributed between the dense-granules and the cytoplasm (5–6 µmoles total ATP in 10^11^ platelets), while most of their ADP is stored in dense-granules (3–3.5 µmoles in 10^11^ platelets). Hence, the global ATP/ADP ratio is 1.5–2.0, whereas in dense-granules, the ATP/ADP ratio is 0.65–0.8 [16,17]. Finally, dense-granules also contain pyrophosphates and polyphosphates. Various studies have demonstrated their importance in the regulation of the coagulation cascade (in particular, via the contact system) and in the inflammatory response [18,19]. As in α-granules, the dense-granule membrane carries receptors, which are not restricted to this membrane, but are also found on the platelet membrane following secretion (CD63, αIIbβ3, GPIb, and LAMP2).

During platelet activation, the dense-granules fuse with the plasma membrane to secrete their contents into the external environment. This phenomenon of exocytosis requires fusion of the granular and cellular membranes through interactions between proteins of the SNARE family, present on the granular membrane (v-SNAREs) and the plasma membrane of platelets (t-SNAREs) [4]. The current model proposes that a complex consisting essentially of three SNAREs (VAMP8, SNAP-23, and STX11) is required to fuse the granular and plasma membranes [20]. Each SNARE protein contains a bi-spiral region composed of 60 amino acids, which catalyze membrane fusion. This interaction of the SNAREs is further regulated by other proteins including chaperones of the Sec1/Munc18 family. RAB proteins (GTPases) are also involved in the secretion process by mediating the docking of dense-granules to the plasma membrane [2].

In the event of abnormalities in the biogenesis, small molecule loading, or secretion of platelet dense-granules, the ability of blood platelets to activate during primary hemostasis is impaired and this can lead to hemorrhagic PFDs, referred to as δ-storage pool diseases. In the following section, we will describe the different forms of these PFDs observed in man.

Key points:

Dense-granules are platelet-specific organelles, which contain small molecules: nucleotides (ATP, ADP), serotonin, and calcium;

Dense-granule secretion is essential to platelet activation;

Dense-granule defects lead to PFDs called δ-storage pool disorders.

## 3. δ-Storage Pool Disorders

As a result of the complexity of platelet dense-granule biogenesis, quantitative or qualitative defects in dense-granules are very heterogeneous. One can distinguish (i) constitutional forms that may be isolated, such as, for example, in the case of abnormalities of the transcription factors GATA1, RUNX1, or Fli1 or of unknown genetic origin, or syndromic associating albinism, ophthalmologic impairment, and immune deficiency (Hermansky–Pudlak, Chediak–Higashi, and Griscelli syndromes) and (ii) acquired forms, which are most often associated with hematological malignancies (myelodysplastic or myeloproliferative syndromes, acute leukemia, and so on) or with auto-immune disease (systemic lupus erythematosus, Sjogren’s syndrome, and so on) [21].

Syndromic forms are more easily recognized clinically owing to the presence of oculocutaneous albinism and immune deficiencies associated with the PFD. Three such pathologies have been described to date: Chediak–Higashi disease, Hermansky–Pudlak syndromes, and Griscelli syndrome.

Chediak–Higashi disease is an autosomal recessive pathology owing to a mutation in the *LYST* gene [22], which leads to abnormalities in vesicular trafficking and the formation of giant granules mainly in melanocytes and leukocytes. The function of the LYST protein (lysosomal trafficking regulator) is poorly known, although the work of Sepulveda et al. [23] suggests that this protein is involved in the correct referral and assembly of late endosomes and lysosomes. The pathology associates a moderately hemorrhagic δ-SPD type PFD with skin hypo-pigmentation and immune deficiency. It generally results in the death of the patients before the age of 10 years, following the development of major lymphohistiocytosis. About forty mutations, homozygous or composite heterozygous, have been reported to date [24].

Hermansky–Pudlak syndromes are autosomal recessive inherited diseases affecting several types of cellular organelles: melanosomes, lysosomes, and platelet dense-granules. Consequently, the phenotype observed in patients is associated with a moderate hemorrhagic syndrome, oculocutaneous albinism, immunodeficiency, and sometimes progressive pulmonary fibrosis. The PFD is characterized by an almost complete absence of dense-granules, which can be highlighted under the electron microscope. These pathologies are owing to mutations in genes encoding proteins of the BLOC-1, 2, and 3 and AP-3 complexes. Eleven genes (*HPS1*, *HPS4* [25], *AP3B1* [26], *HPS3*, *HPS5*, *HPS6* [27], *DTNBP1* [28], *BLOC1S3* [29], BLOC1S5 [30], *BLOC1S6* [31], and *AP3D1* [32,33]) have been found to be involved in these syndromes in 715 patients, with the severity of the disease depending on the gene affected and the type of mutation [34]. In particular, syndromes resulting from *HPS1* mutations are considered to be the most serious forms owing to the high frequency of related pulmonary fibrosis.

Finally, mutations in the gene coding for the GTPase RAB27 are responsible for Griscelli syndrome [35]. This autosomal recessive inherited disorder is characterized by a hemorrhagic syndrome linked to a decrease in the number of platelet dense-granules, albinism, neurological impairment, and immune deficiency [36].

Non-syndromic δ-SPDs are PFDs whose cutaneous and mucosal hemorrhagic phenotype is often considered to be moderate under non-traumatic conditions. However, in the event of surgery or trauma, there is a significant risk of major bleeding, sometimes life-threatening, in these patients. Non-syndromic δ-SPD may be owing to the absence or a reduction in the number of dense-granules, a qualitative defect in these organelles, or a combination of these two defects [4]. Among the qualitative defect, isolated ADP defect, isolated serotonin defect and combined serotonin, and ADP deficiency are described. In all cases, the pathophysiological and molecular mechanisms leading to these isolated platelet disorders are currently uncertain. Moreover, in some patients, the decrease in the number of dense-granules is accompanied by a decrease in the number of α-granules and gives rise to PFD called αδ-SPD, which is thought to be linked to platelet degranulation in the absence of platelet activation [37]. Recently, combined αδ-SPD can be associated with GFI1B sequence variations [38].

According to the experience of our laboratory, the majority of non-syndromic δ-SPD cases appear to result from a deficiency in dense-granules nucleotides, especially in ADP. In this case, deficiencies in nucleotide transporters such as MRP4 are suspected to be responsible for the pathology. Thus, an abnormal localization of the MRP4 protein has been demonstrated in some patients with δ-SPD [39]. However, no team has yet been able to detect a mutation in the *ABCC4* gene encoding MRP4. In conclusion, no gene has to date been identified as causative of non-syndromic δ-SPD in man.

On account of the heterogeneous biological characteristics of non-syndromic δ-SPD, the diagnosis of this pathology remains difficult and poorly standardized [40]. Hence, the frequency of the disease remains unknown, although some studies suggest that non-syndromic δ-SPD may be a particularly frequent congenital PFD [41,42]. As an example, in a retrospective study conducted in France in 2017 by the CRPP (French reference center for platelet diseases), it was shown, after analysis of the “care cards” issued to patients, that among 283 patients with PFDs, 25% presented a dense-granule defect, the second cause of platelet disease in this cohort (poster in International Society of Thrombosis and Haemostasis (ISTH) 2017 congress [43]). Given the probable high frequency of this kind of PFD, a precise definition of the disease and technical harmonization of the biological tests to be carried out to diagnose δ-SPD would seem indispensable.

The diagnosis of δ-SPD, in particular of its non-syndromic forms, represents a real technical challenge, as the laboratories must have specialized and complex tools available in order to obtain an accurate result. These specialized tools, mandatory for the diagnosis of δ-SPD, will be described in the following section.

Key points:

δ-SPDs can be syndromic or non-syndromic diseases;

Syndromic forms associate oculocutaneous albinism, immune deficiency, and bleeding;

For non-syndromic forms, the genes responsible for the disease remain unknown.

## 4. Tools for the Diagnosis of δ-SPD

Numerous biological tests exploring dense-granules are available, but few laboratories use them in routine practice because many are highly specialized and not standardized. Thus, in an international survey conducted by the ISTH, more than half of the laboratories interviewed reported that they did not evaluate platelet granules [44]. In addition, there are no validated recommendations concerning the decision tree and the prioritization of tests to achieve an accurate diagnosis [40]. Finally, the platelet function analyser (PFA), which is widely used in haemostasis labs to detect primary haemostasis defects, remains poorly sensitive to screen patient for δ-SPD [45]. It may nevertheless be said that the diagnosis of these PFDs is based on functional abnormalities, which can be objectively, although inconsistently, detected in aggregation tests, before looking for a quantitative defect in dense-granules by electron microscopy, and on the detection of deficiencies in small molecules contained in these granules. Tests measuring the secretion of dense-granules by lumi-aggregometry or flow cytometry may also be used.

### 4.1. Aggregation Tests

**Figure 1:**
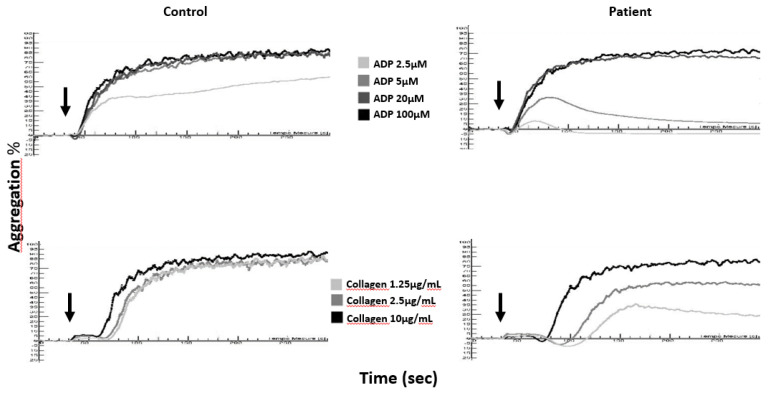
Aggregation curves in citrated platelet-rich plasma (cPRP) of a δ-SPD patient and a healthy control (Control) in response to ADP (2.5, 5, 20, and 100 µM) and collagen (1.25, 2.5, and 10 µg/mL); arrows denote the times of addition of the agonists to cPRP.

To date, light transmission aggregometry (LTA) remains the reference test for the diagnosis of PFDs. Aggregation tests can be performed either with a citrated platelet-rich plasma (cPRP) or with a washed platelet suspension. A cPRP is obtained directly after centrifugation of whole blood collected in 0.109 or 0.129 M sodium citrate tubes, while a washed platelets suspension is obtained after centrifugation of whole collected in ACD anticoagulant (acid citrate dextrose) and resuspension of the platelets in Tyrode-albumin buffer [46]. Abnormalities in platelet aggregation are often moderate in dense-granule deficiencies, and approximately 25% of patients with δ-SPD display no platelet function defect [47] when platelet function studies are performed in cPRP. Nonetheless, the typical cPRP aggregation patterns observed in δ-SPD patients are described in Figure 1. The main typical anomalies are as follows:

The absence of a second wave in ADP (2.5 and 5 µM)-induced platelet aggregation;

A decrease in the maximum amplitude of collagen-induced aggregation together with an increase in the lag time, especially when a low concentration of collagen is used (1.25 µg/mL) [48];

Arachidonic acid-induced platelet aggregation is normal or decreased depending on the intensity of the ADP-induced aggregation defect in the same sample [49];

Although widely used, epinephrine-induced aggregation should not be recommended to detect δ-SPD [50].

**Figure 2:**
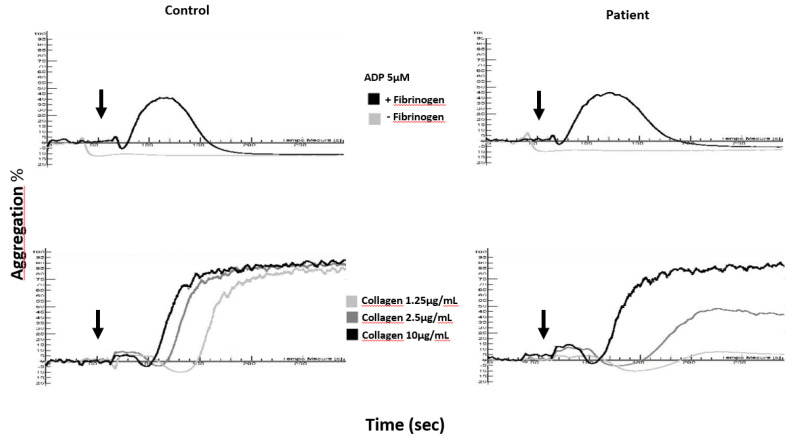
Aggregation curves in a washed platelet suspension of a delta-storage pool disease (δ-SPD) patient and a healthy control (Control) in response to ADP (5 µM) and collagen (1.25, 2.5 and 10 µg/mL); arrows denote the times of addition of the agonists to washed platelets.

In a washed platelet suspension (Figure 2), one does not observe the same abnormalities as in cPRP after stimulation of platelets with ADP, whatever the concentration of agonist used. This is because ADP does not induce platelet degranulation or thromboxane A2 generation in the presence of 2 mM calcium [46,51,52] and aggregation is by definition reversible. Unlike ADP-induced aggregation which remains normal, collagen (1.25 and 2.5 µg/mL)-induced aggregation generally displays more pronounced anomalies than those observed in cPRP. Citrated PRP may contain trace amounts of release nucleotides and thrombin [50], which can potentiate platelet activation and thus may hide moderate platelet function defect such as those observed in δ-SPD. Whatever it is, LTA alone is not sufficient to establish a diagnosis of δ-SPD.

In the case of suspicion of δ-SPD, the aim of the complementary tests to be carried out is then to demonstrate a reduction in the quantity of small molecules (ADP and/or serotonin) contained in the dense granules, associated or not with the presence of fewer or no dense-granules, as observed by electron microscopy. A study of platelet secretion (by flow cytometry or lumi-aggregometry) may allow one to complete the characterization of the functional defect in aggregation and possibly highlight an anomaly of the secretory capacity of the platelets in the absence of a granular deficiency.

### 4.2. Ultrastructural Evaluation of the Dense Granules Using a Whole Platelet Mount

The first publication to mention the use of “unfixed unstained whole platelet” transmission electron microscopy and to describe the presence of round or tadpole-shaped electron-dense bodies is believed to be that of Brian S. Bull in 1966 [53]. The localization of platelet serotonin in these naturally electron-dense granules was suggested in the following years (reviewed by White in 1969 [54]). The role of calcium in this natural opacity to electrons was not demonstrated until the following decade [55], together with the colocalization of calcium and phosphorus [56], with the colocalization of serotonin and ATP having been mentioned earlier in 1958 by Born [57]. Pyrophosphates [58] and polyphosphates [59] were more recently identified as participating in the electron opacity of these granules, which bear a strong resemblance to the acidocalcisomes of bacteria and unicellular eukaryotes. Similar electron-dense nanoparticles have been synthesized in controlled precipitations of soluble polyphosphates with calcium ± magnesium [60]. Polyphosphates are essential calcium chelators and reservoirs of ATP in the presence of ADP and have a potential strong buffering capacity in the presence of serotonin [61]; their multiple roles in thrombosis, hemostasis, and inflammation have been extensively reviewed by Mailer et al. [62] and illustrated by Baker et al. [63]. By remaining firmly attached to the platelet after its release, a dense particle is able to activate the coagulation contact pathway, which is not the case for short platelet polyphosphate polymers in solution [64].

#### 4.2.1. Sample Preparation

**Figure 3:**
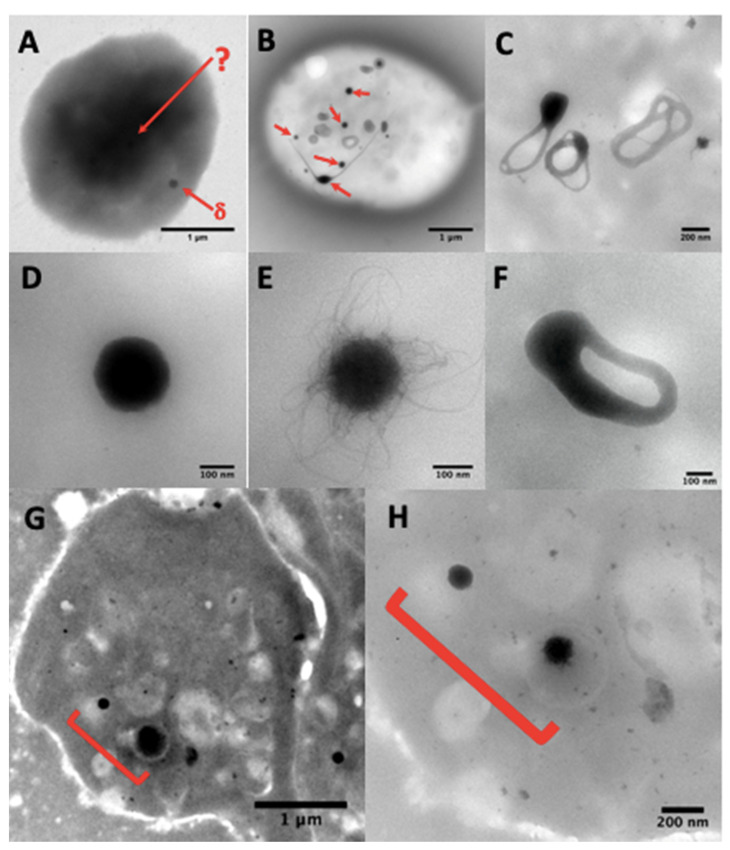
(**A**) A whole platelet mount from a platelet suspension incubated in 1.25% glutaraldehyde phosphate buffer for one hour. The use of aldehyde fixatives, even very briefly, is not recommended and paraformaldehyde treatment gives similar results; the platelet background becomes so dark that only some peripheral granules (δ-arrow) are sometimes visible, but no granules in the platelet center (δ-arrow). (**B**) dense-granules (red arrows). A simple brief wash in pure water of native platelets on a formvar film is sufficient to obtain electron-translucent cells where the dense-granules are perfectly identifiable. These granules are classically smooth (**D**) or with filaments (**E**), but are sometimes observable in restructuring phases as annular forms (**C**) that split into long extensions (**B**, bottom left center), possibly also in a division phase (**F**), where two nodules are visible, which will probably evolve into two well separated dense-granules. The camera in automatic contrast adjustment mode may make the identification of dense-granules doubtful. The quickest solution is to change the magnification rather than the long contrast-brightness-gamma settings; an element looking like a veil at Mag 20 k (**G**) will be better identifiable at Mag 80 k (**H**).

Dense-granule contents should be determined using unfixed whole native platelet mounts prepared and examined as described by White [65] on formvar ultra-thin (UL 5–6 nm) 200 mesh Cugrids (Electron Microscopy Sciences, Hatfield, Pennsylvania). Correct counting of the granules is not easy or impossible in aldehyde-fixed platelets (Figure 3A) as the treatment makes the platelet background very dark and the contrast of the dense elements becomes too weak. Hydrophilization by glow discharge and/or the use of carbonated formvar grids present no advantages. The carbon film is fragile and unstable over time; when cracked, it does not facilitate the visualization of platelets with a camera in automatic contrast adjustment mode.

Citrated PRP is isolated from blood collected into a CPDA1 tube (Sarstedt, Marney, France) by centrifugation at 150 G for 10 min, or less in the case of severe thrombocytopenia. Sodium citrate [66] or acid citrate dextrose (ACD) [67] are also used in other standard published methods. The blood is processed within 4 h of collection, as for other platelet function tests such as cytometry or aggregometry, according to the ISTH recommendations for platelets [49]. Routinely, five formvar grids from various batches are incubated for 1 to 5 min in small drops of PRP deposed on a parafilm. The use of different batches of grids is recommended because many irregularities in thickness and many cracks, which are frequent in a batch, make it more difficult to count the dense granules.

The grids are then briefly washed in a drop of pure water, with excess water being rapidly removed by capillarity at the edge of a tissue paper, after which they are dried by slow, wide movements in the air. Observation under the microscope can be done immediately and is usually performed during the following week. The grids must be stored at room temperature, protected from dust, light, and humidity. The grids can be easily prepared in a few minutes by the technicians of a hemostasis laboratory not specialized in electron microscopy. Sending the grids at room temperature to a microscopy center is also much easier than sending a tube of blood under questionable conditions of transport time and temperature. The patient’s external blood center only requires a stock of formvar grids, good fine forceps, pure water, tissue paper, and parafilm.

#### 4.2.2. Ultrastructural Examination of Dense-Granules

Granules are usually counted at Mag 20–40 k, or sometimes identified at Mag 80–150 k in the case of doubtful contrast in poorly granulated platelets (Figure 3G,H), using a JEOL JEM1400 transmission electron microscope at 80–100 kev, equipped with a penta-holder, a Gatan Orius 600 camera, and Digital Micrograph software (Lyon Bio Image, Centre d’Imagerie Quantitative de Lyon Est, France).

Normal dense-granules are uniformly dark and round, perfectly smooth, or with hairy processes, or can look like a torus (Figure 3B–E), sometimes with one or more tails (Figure 3B); round forms have a diameter of 161 ± 42 nm (mean ± 1SD, *n* = 713 in 40 samples). Nonetheless, some patients having a bleeding history, but a normal average of dense granules had a small delta storage pool volume called micro-granular storage pool deficiency (delta-MGSPD) [68].

**Figure 4:**
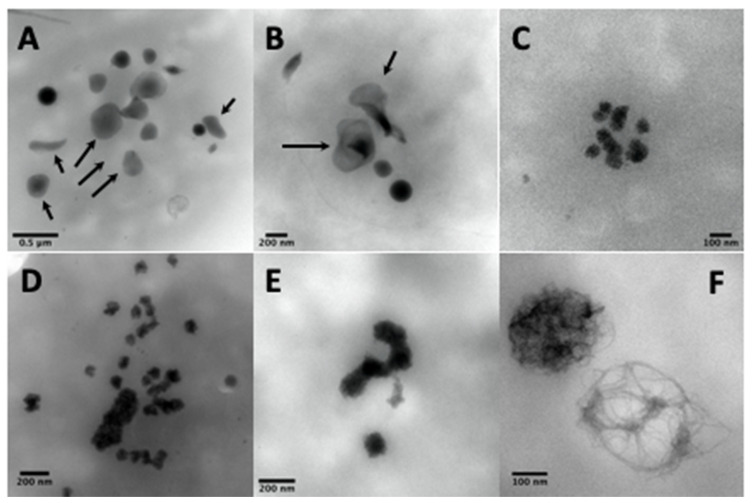
The electron-dense elements not counted as dense-granules are calcium- and phosphorus-poor veils [67] (**A**,**B**, arrows), clusters of spongy elements (**C**), also present in the form of chains or larger grains (**D**,**E**), which are found in abundance and bigger in STIM1–York–Stormorken pathologies and are rich in calcium and phosphorus [70], while others are poorer in calcium (**F**), which could represent immature dense-granules (possibly polyphosphate filaments without calcium accumulation, found in non-identified cases of delta deficiency, but not in Hermansky–Pudlak syndrome).

Other electron-dense structures not counted as dense-granules are large, thin semi-opaque veils (Figure 4A,B); poor in phosphorus and calcium [67]; with fuzzy or spongy elements (Figure 4D), which occur very large and numerous in STIM1–York–Stormorken syndromes [69] and clusters or chains of small beads (Figure 4C–E). The latter have a near-delta composition in calcium and phosphorus, but a lower P/Ca ratio for the chains, as determined by EDXA (Energy-dispersive X-ray spectroscopy). Chains and fuzzy balls are not released after thrombin stimulation [70] and could represent a local accumulation of calcium, phosphorus, and other materials that failed to be normally incorporated into mature dense granules. In our laboratory, other structures with the appearance of balls of filaments were found in a family with dense-granule deficiency (Figure 4F). These balls display a size close to that of classical dense-granules, but with a high P/Ca ratio, and might possibly represent an aborted form appearing during dense-granule formation owing to a lack of calcium intake. Present in small numbers in platelets, their release in response to thrombin has not yet been evaluated and no defect has been found to date in the set of genes currently targeted in the platelets of this family.

The whole platelet method in transmission electron microscopy requires some training in the counting of dense-granules. Moreover, the number of platelets totally devoid of dense-granules is sometimes difficult to evaluate owing to a lack of reference marks for their identification on a formvar film of variable quality. A grid rinsed too long in water or a problem related to an unusual phenomenon of surface quality or hydrophobia of the formvar can produce spherical platelets; the focus becomes delicate and these platelets can be counted as containing no granules. Hence, it is advisable to look at several areas on all the grids before starting to count the number of granules per platelet. A control exercise in the recognition of dense-granules is proposed twice a year by the NASCOLA-ECAT with images provided by Dr. Dong Chen of the Mayo Foundation for Medical Education and Research.

#### 4.2.3. Counting of Dense-Granules

**Figure 5:**
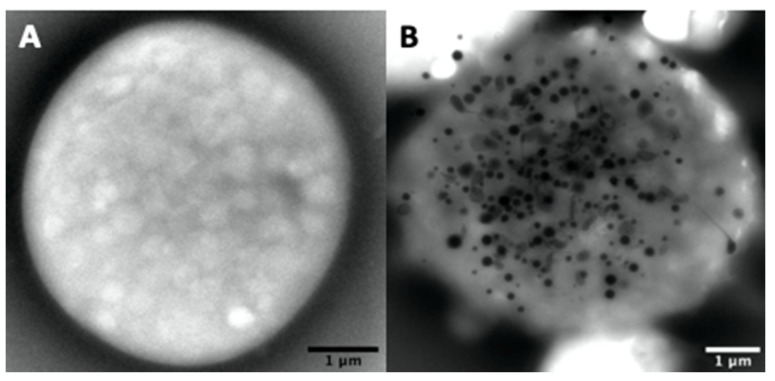
Examples of a platelet totally devoid of electron-dense material (**A**) like those found in Hermansky–Pudlak syndrome (HPS) patients and a macro-platelet of a gray platelet syndrome (GPS) patient (**B**) with 155 dense-granules (mean 14 ± 26/platelet, 17% of platelets with >20 dense-granules).

The number of dense-granules per platelet ranges varies widely, from absolute zero in Hermansky–Pudlak syndrome (HPS) to over 150 in the macro-thrombocytes of a gray platelet syndrome (GPS), for example (Figure 5).

In the literature, the range of distribution is also relatively broad in healthy control subjects, with the current reference intervals being highly variable according to the laboratories; for example, >3.68 dense-granules/platelet (mean ± 3SD) [71]; 1.95–4.37, *n* = 40 (19 males and 21 females) in young people (median age 10 years) [72]; 1.2–4.0, *n* = 113 (65 males and 48 females) in adults [67]; or intervals of 4.9–8.2 for 60 males and 4.9–8.8 for 66 females (2.5–97.5 percentile ranges) [66]. The evaluation by electron cryotomography in 30 platelets from six healthy subjects was 5.5 ± 1.9 dense-granules/platelet [73], while that of Eckly et al. using focused ion beam-scanning electron microscopy (FIB-SEM) was 8.0 ± 4.2 dense-granules/platelet in 49 platelets reconstituted in 3D [74] and Pokrovskaya et al. using serial block face-scanning electron microscopy (SBF-SEM) found 5.5 ± 2.5 dense-granules/platelet in 30 platelets from three donors, with 10 platelets studied for each donor [75] (mean values ± 1SD).

**Figure 6:**
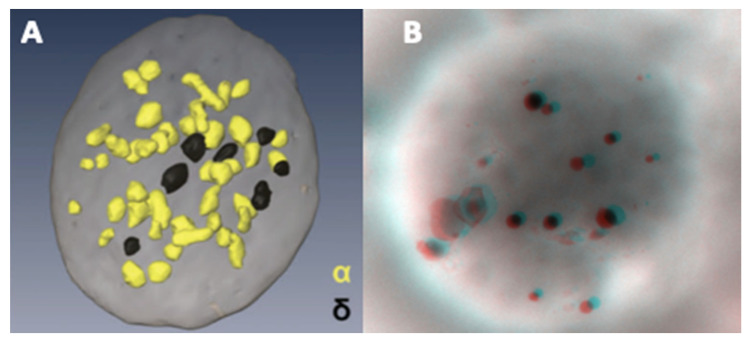
Using conventional electron microscopy with 70 nm thick sections, the probability of observing a dense granule is low. An alternative is the reconstruction of serial sections. The rapid, more, or less automated method of focused ion beam-scanning electron microscopy (FIB-SEM) makes it possible to observe the spatial distribution of α- (yellow) and dense-granules (black) in a platelet. (**A**) data from Eckly et al. published in [76] (part of Figure 6B, image obtained from the Haematologica Journal website http://www.haematologica.org). A stereo image of an entire platelet on formvar can be obtained from two tilt images taken at ±7°. (**B**) Anaglyph reconstructed with ImageJ (V1.8 NIH, USA) Two Shot Anaglyph software (V2.9.5, Sandy Knoll Software, USA); the stereo effect is visible with red-cyan glasses.

An example of an FIB-SEM reconstitution showing the relative abundance of α- versus dense-granules is presented in Figure 6A [76], alongside an anaglyph image (Figure 6B) obtained from two tilts of a whole platelet mount; the latter requires red-cyan glasses to see the stereo effect. Our reference value determined according to the method of Brunet et al. [66], without taking into account gender, is 4.14–7.74 in 54 subjects, that is, 5400 platelets, as the 2.5–97.5 percentile range of the mean/subject. Routinely, the results of the examinations requested for patients are derived from the granule counts in 100 platelets. A recent study using LEAN methodology suggests to only count delta in 20 platelets in case when the average is >3 and in 100 platelets when the average is <3 [77]. These counts are also highly variable in the literature. Statistics are available for evaluations of 30 [66] to 200 [67] platelets and all these values are summarized in Table 1. Considering the range of published reference intervals, each observer needs to establish his own values.

#### 4.2.4. Distribution Statistics

The average result alone does not always allow one to account for the distribution of granules in platelets or to distinguish the presence of platelet subpopulations, as described in cases of mutations in *GFI1B* and *RUNX1* [78]. A scatter plot of hundreds or thousands of values is not very practical for comparisons of small variations; a histogram would be better, but too many overlays make it less visual. The cumulative distribution frequency or Q-plot representation would thus seem the most practical to visualize results with large deviations and permits at a glance the determination of a percentage at the desired threshold.

**Figure 7:**
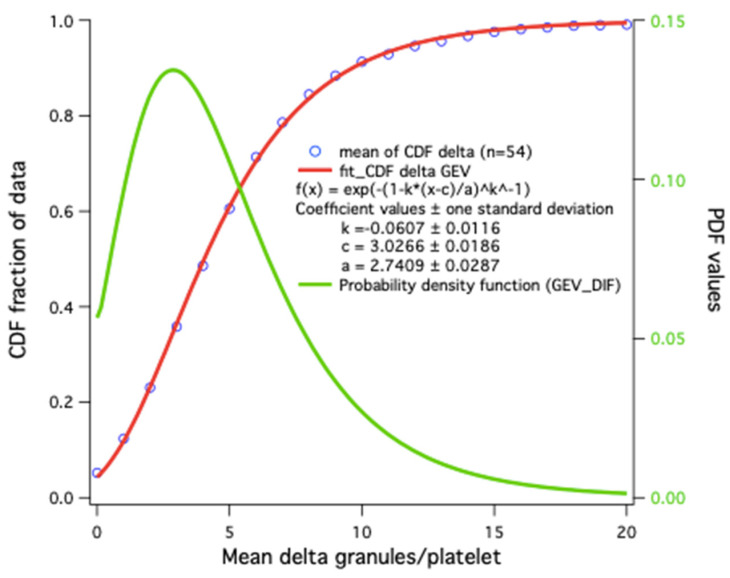
Distribution model of the number of dense-granules/platelet: cumulative distribution frequency (CDF) averages in 54 controls (blue circles). The simplest fit is a generalized extreme value (GEV) distribution function (red); the corresponding probability density obtained by CDF derivative (Igor Wavemetrics) is shown in green.

**Figure 8:**
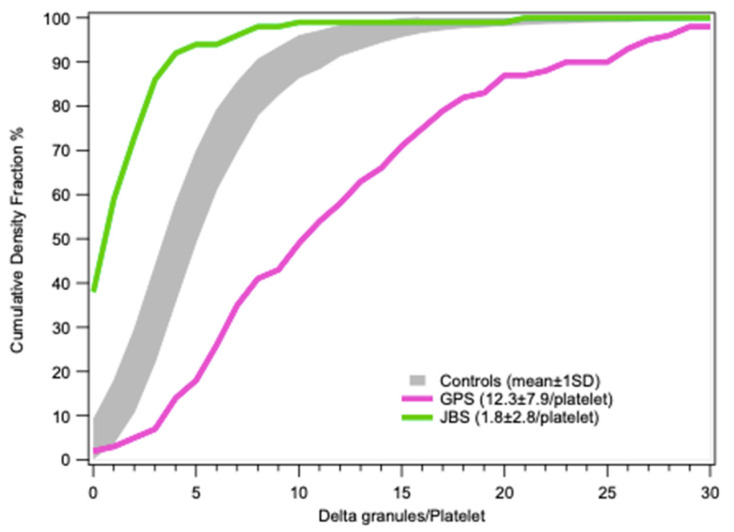
Cumulative frequencies of dense-granule distributions in platelets (mean ± 1SD) in controls (gray, *n* = 54) and two cases of macrothombocytopenia with similar platelet size distributions, a patient with GPS whose platelet dense granule content may be considered to be normal and a patient with Jacobsen syndrome (JBS) having a severe deficit.

Statistical results for platelet dense-granules are shown in Figure 7. A Q-plot of the number of dense-granules/platelet in our healthy controls fits a generalized extreme value distribution. This is the simplest fit found; *p*-values for the distributions in patient platelets versus controls are derived using a Kolmogorov–Smirnov test with the Marsaglia method in R for borderline patient distributions. Cumulative dense-granule distributions in the cases of gray platelet syndrome (GPS) and Jacobsen syndrome (JBS) as compared with our controls are illustrated in Figure 8. The important differences in distribution do not require any statistical test to appreciate their significance. These two patients with GPS and JBS have macro-platelets of similar size; the GPS platelets contain dense-granules in proportion to their size, and the JBS platelets may thus be considered to have a very severe deficit in dense-granules.

The values obtained in children are difficult to interpret [72] and it is well recommended to repeat the evaluation at a more advanced age when subnormal counts are recorded [79]. The bleeding scores and platelet dense granule contents have been found to display a significantly weak correlation in a pediatric population [80].

### 4.3. Other Studies of Granular Content

#### 4.3.1. ATP and ADP Contents

The determination of platelet nucleotides (ATP and ADP) by luminescence or high-performance liquid chromatography (HPLC) on an anion exchange column is relatively uncommon, although these are specific and reproducible tools to characterize abnormalities of platelet organelles. As ADP is predominantly concentrated in the dense-granules, a total ATP/ADP ratio exceeding 4 (normal laboratory values: 1.1–2.2) will favor a dense-granule defect. A nucleotide assay in combination with a platelet serotonin assay by ELISA (enzyme-linked immunosorbent assay) or HPLC(high liquid performance chromatography) is still not widely used in hemostasis laboratories. However, in association with the quantification of dense-granules by electron microscopy, this technique makes it possible to diagnose δ-SPD linked to a qualitative or quantitative deficiency in platelet dense-granules [40].

#### 4.3.2. Serotonin Content

One of the most common methods classically used to determine platelet serotonin content is HPLC with electrochemical detection following platelet lysis with perchloric acid in the presence of an internal standard such as methylserotonin [81,82]. Therapy with selective serotonin reuptake inhibitors interferes with the evaluation [83,84].

#### 4.3.3. Fluorescence Microscopy

Mepacrine Labeling

A technical feat at the time, correlative mepacrine fluorescence and whole platelet mount electron microscopy of dense bodies was first performed by Skaer et al. in 1981 [85]. These authors demonstrated the specificity of mepacrine for dense-granules, without any lysosome labeling in platelets.

Polyphosphates/DAPI (4’,6-Diamidino-2-Phenylindole)

The binding of DAPI to polyphosphates shifts its classical peak emission wavelength with nucleic acids from 475 nm (blue) to 525 nm (yellow-green) following excitation at 360 nm [59], which allows the use of DAPI to detect polyphosphates in vitro and in live polyphosphate accumulating organelles.

Proteins of Dense-Granules

Lamp 1, Lamp 2, and CD63 labeling can help to identify δ-SPD on a smear [86,87]. Using high resolution microscopy, a two-color image method has been proposed by Westmorland et al. [88], which employs sequential image acquisition during excitation of the sample with laser light at 488 nm for CD63 and 561 nm for tubulin in order to delimit the exact individual platelet areas. This method makes it possible to orient the diagnosis towards a dense granule deficit, but the labeling is not specific for dense-granules, as shown by comparison with the counting of the granules by whole mount electron microscopy in the same control and HPS samples [88].

### 4.4. Secretion Tests

#### 4.4.1. Uptake and Release of Radio-Labeled Serotonin

The study of the incorporation and secretion of radio-labeled serotonin [89] is still considered to be the reference test to evaluate platelet secretion capacity. When incubated with platelets, tritium- or carbon-14-labeled serotonin is rapidly incorporated into the dense-granules. The cells are then stimulated with agonists such as collagen or thrombin and the amount of serotonin released into the medium can be quantified using a suitable counter. This technique can be employed to assess both the capacity of platelets to capture serotonin and incorporate it into dense-granules and their ability to secrete it [40]. In the case of δ-SPD with an absence of dense-granules, the uptake and secretion of serotonin will be abolished. Although this is the reference method to identify a secretion defect, it does not allow one to differentiate between a quantitative deficiency in dense-granules and an intrinsic platelet secretion defect related to impaired signaling.

#### 4.4.2. Chemical or Biochemical Measurement of Serotonin Release

After platelet activation, serotonin can also be measured in the platelet supernatant using a commercial ELISA kit, or by HPLC with a fluorometer (excitation 285 nm–emission 350 nm) [90] or an electrochemical detector, usually a glass/carbon electrode with a Ag/AgCl reference electrode [91]. Thus, electrochemical detection or amperometry has been used with a carbon-fiber microelectrode to measure the release of serotonin from a single platelet [92] and the method has been adapted to follow the kinetics of serotonin release simultaneously during platelet aggregation [93]. This technique represents an alternative to detection of the release of ATP (§ 4.4.6) and especially to the measurement of the release of radio-labeled serotonin (§ 4.4.1).

#### 4.4.3. Mepacrine Flow Cytometry

The “mepacrine test” permits an evaluation of the incorporation and secretion capacities of platelets. Mepacrine [94], a fluorescent marker derived from acridine orange that binds adenosine nucleotides, is incorporated into platelets via the same transporter as serotonin (SERT). The mepacrine captured by the dense-granules is then secreted upon stimulation of the cells with various agonists. Platelet fluorescence can thus be quantified by flow cytometry before and after stimulation. However, although this method can be used to evaluate the presence and the secretion of dense-granules, it has at least two drawbacks: the experiment has to be done within 6 h after blood collection and the mepacrine test has only a moderate sensibility (76%) and specificity (83%) for the detection of δ-SPD [95]. Therefore, this test should not be employed in isolation for the diagnosis of δ-SPD.

#### 4.4.4. Polyphosphate Release

Using excitation at 415 nm and as little as 25 ng/mL of polyphosphate, the fluorescence of the DAPI–polyphosphate complex can be detected at a higher wavelength (550 nm) [96], without interference from DNA or RNA labeling or ATP and ADP. This method has been used to measure polyphosphate release from platelets after extraction–purification procedures [97]. It is nevertheless unsuitable to count dense-granules under a microscope owing to the non-specific autofluorescence of other platelet components and the fast quenching of DAPI. The rapid photobleaching of DAPI at alkaline pH (10.5) makes it possible to produce negative images of electrophoresis gels and considerably increases the detection limits of polyphosphates [98]. The use of live-cell imaging under flow, the impermeant dye SYTOX Orange, and Alexa488-conjugated exopolyphosphatase have enabled the identification of polyphosphate binding domains (PPX Δ12) and the measurement of polyphosphate release [64] during platelet adhesion/activation.

#### 4.4.5. Flow Cytometry of CD63 Exposure

As mentioned above, granulophysin (CD63) is a transmembrane protein found on dense-granules and lysosomes. Its expression on the platelet surface before and after platelet activation is easily measured by flow cytometry. In the case of a dense-granule deficiency, CD63 exposure after activation of platelets with a thrombin receptor activator peptide (TRAP 50 µM) will be severely decreased. However, this test does not permit one to separate a secretion defect from a dense-granule deficiency. Another limitation in its use is that CD63 is also expressed on the surface of lysosomes and is not strictly specific to dense-granules.

#### 4.4.6. Lumi-Aggregometry

The fastest and most widespread technique used in specialized hematology laboratories for platelet exploration is lumi-aggregometry. The principle of this method exploits the simultaneous measurement of platelet aggregation by light transmission or impedance and ATP release into the extracellular medium throughout the aggregation process [99,100]. The secreted ATP is converted into luciferin adenylate in the presence of luciferin and magnesium. Luciferin adenylate is then converted by oxygen into oxyluciferin and produces easily quantifiable light. This technique makes it possible to highlight secretion anomalies in individual responses to collagen, ADP, epinephrine, U46619, or other agonists. However, lumi-aggregometry suffers from a lack of sensitivity and reproducibility in detecting δ-SPD patients [101].

### 4.5. Genetic Studies

Genetic analyses must be systematically proposed in the event of suspicion of a deficit in dense-granules of constitutive origin. Several genes responsible for Hermansky–Pudlak, Chediak–Higashi, and Griscelli syndromes have been sequenced, while in forms associated with thrombocytopenia, molecular abnormalities may involve transcription factors such as RUNX1 or FLI1 [76,102,103]. Nonetheless, in the majority of cases, isolated forms of dense-granule deficiency remain without genetic diagnosis. The possibility of simultaneously exploring several genes (gene panel, whole exome, whole genome) using new high-throughput sequencing techniques should make it possible to identify new genes involved in these deficits in the coming years.

Key points:

LTA is the gold standard to study platelet functions, but remains poorly sensitive for detecting δ-SPD (except if LTA is performed with a washed platelets suspension);

Secretion tests are helpful to suspect δ-SPD;

Electron microscopy (whole mount) is the gold standard to quantify dense-granules;

Study of granular content is essential to highlight qualitative defects in dense-granules.

## 5. Diagnosis Strategy and Clinical Management of δ-SPD Patients

### 5.1. Diagnosis Strategy of δ-SPD Patients

Diagnosis and management of bleeding risk in δ-SPD patient remain complex mainly in non-syndromic forms of the disease. Because the diagnosis relies on the use of specialized technics such as electron microscopy, a collaboration between physicians, routine haemostasis clinical labs, and research labs is recommended.

In syndromic forms, the combination of oculocutaneous depigmentation, susceptibility to infection owing to an immune deficiency, and a moderate hemorrhagic syndrome should result in a request for a study of platelet function using aggregation tests. Whatever the aggregation anomalies observed, the determination of granule contents and quantification of platelet dense-granules should complement the initial tests performed. An absence of dense-granules, associated with serotonin deficiency and an ATP/ADP ratio of more than 4, allows the diagnosis to be oriented towards Chediak–Higashi disease, Hermansky–Pudlak syndrome, or Griscelli syndrome. The clinical presentation is sometimes unclear, as in the case of BLOC2 complex abnormalities (*HPS3*, *HPS5*, and *HPS6* genes) [34,104,105], and sequencing of the *HPS*, *LYST*, and *RAB27* genes is then the final step to an accurate diagnosis.

In non-syndromic forms of δ-SPD, platelet function studies in cPRP are poorly sensitive to highlight dense-granule or secretion defects, especially if thrombocytopenia is also present. In our experience, function studies performed with washed platelets are more accurate to identify dense-granule deficiencies. Therefore, an analysis of platelet dense-granules should be performed in all patients displaying abnormalities of primary hemostasis after exclusion of the most common PFDs, even if platelet aggregation shows no abnormalities. Flow cytometry tests (CD63 exposure, mepacrine test) are also useful tools for screening patients [95,106]. As for syndromic forms of δ-SPD, the biological exploration of dense-granules should include the determination of granule contents and quantification of dense-granules by electron microscopy. If dense-granule deficiency is confirmed, in the presence of thrombocytopenia, a study of the *RUNX1*, *FLI1*, and *GATA1* genes is recommended as a first-line complementary measure. In the absence of thrombocytopenia, unfortunately no gene has yet been shown to be responsible for the pathology. Hence, patients suffering from this pathology should be included in large studies using high-throughput sequencing techniques to identify the currently unknown genes involved.

### 5.2. Bleeding Risk and Clinical Management of δ-SPD Patients

Patients with any type of δ-SPD suffer from moderate bleeding syndromes with little disruption of daily life in general. Some recent retrospective clinical surveys have confirmed that patients with δ-SPD have a moderately high (mean = 5) ISTH-BAT (International Society of Thrombosis and Haemostasis-Bleeding Assessment Tool) hemorrhagic score [107] as compared with patients with suspected PFDs (mean = 8) [108]. The moderate risk of bleeding, even in a standard surgical situation, was further confirmed by the multicenter study of Gresele et al. [109] conducted in 423 patients, including 238 having PFDs (17 patients with δ-SPD). Interestingly, minor atypical bleeding was observed by Civaschi et al. [110] in pregnant women with δ-SPD (*n* = 20) and two cases were reported to need blood transfusion.

Hence, in the case of surgery, prophylactic platelet transfusion is not recommended for patients with δ-SPD. Prophylactic and post-operative treatment using tranexamic acid and desmopressin should be preferred as long as the bleeding risk is limited. Because platelet transfusion is not mandatory to manage moderate bleeding for these patients and because it may lead to platelet refractoriness, platelet transfusion should be reserved for massive bleeding events and for bleeding that is not controlled by classical adjuvant therapy [111]. Activated recombinant human factor VII can also help to stop massive bleeding in the most complicated cases.

Finally, in order to limit the risk of bleeding in these patients, the use of any other drug having an impact on platelet physiology should be discussed in particular antidepressants such as serotonin reuptake inhibitors and platelet anti-platelet therapies in the case of thrombotic diseases. This approach avoids the deleterious effect of a combination of dense-granule deficiency and blockage of other platelet activation pathways essential for the formation of a platelet aggregate, the first stage of hemostasis, which stops bleeding.

The prevalence of δ-SPD is probably underestimated. Indeed, some unexplained bleeding during or after surgery may be caused by non-syndromic types of δ-SPD, which have never been diagnosed in the majority of patients. This is probably owing to the fact that the diagnostic tools are not standardized, sometimes very expensive, and complex to implement routinely in hemostasis laboratories. The harmonization of diagnostic methods and sharing of expensive and sophisticated tools like electron microscopes should be a valuable strategy to improve the identification of δ-SPD patients, in order to search for the genes responsible for the non-syndromic forms of the disease, and thus provide appropriate treatment to limit bleeding episodes in these patients.

Key points:

Accurate diagnosis of δ-SPD needs interaction between routine haemostasis and research labs;

Electron microscopy and δ-granular content study are mandatory to diagnose δ-SPD, especially the non-syndromic forms;

In case of bleeding, the transfusion of platelet concentrates should be used only when tranexamic acid and/or desmopressin are ineffective.

## Figures and Tables

**Table 1:** Dense-granules (DG)/platelet (reference interval 2.5–97.5 percentile or mean ± 1SD).

Method	DG/plt	Number of Subjects	Number of plts/Subject	Age Range	Anticoagulant	PRP Preparation	Reference
WP-TEM	>3.68 (m − 3SD)	NI	NI	NI	ACD	100 g 15 min <48 h	Gunning 2000-20 [68,71]
WP-TEM	1.95–4.37	*n* = 40 (19M + 21F)	100	2 m–21 year	ACD	57 g 20 min	Sorokin 2016 [72]
WP-TEM	1.2–4.0	*n* = 113 (65M + 48F)	100–200	18–70 year	ACD-A or -B	200 g 20 min	Chen 2018 [67]
WT-TEM	>3	*n* = 300	20(>3)–100(<3)	6 w–21 year	EDTA	100 g * 6 min <72 h	Asher 2020 [77]
WP-TEM	4.9–8.2	*n* = 60 M	30–50	15–64 year	Na citrate	115 g 15 min <4 h	Brunet 2018 [66]
WP-TEM	4.9–8.8	*n* = 66 F	30–50	15–64 year	Na citrate	115 g 15 min <4 h	Brunet 2018 [66]
WP-TEM	4.14–7.74	*n* = 54	100	18–70 year	CPDA1	150 g 10 min <4 h	Pennamen 2020 [30]
CT-TEM	5.5 ± 1.9	*n* = 6 F	5	NI	Na citrate	150 g 20 min <1 h	Wang 2015 [73]
FIB-SEM	8.0 ± 4.2	49 plts	NI	NI	ACD	250 g 15 min **	Eckly 2016 [74]
SBF-SEM	5.5 ± 2.5	*n* = 3	10	NI	Na citrate	NI (after fixation)	Pokrovskaya 2020 [75]

M: male; F: female; NI: not indicated; w: week; m: month; y: year; h: hours; WP: whole platelet; TEM: transmission electron microscopy; CT: cryotomography; FIB: focused ion beam; SEM: scanning electron microscopy; SBF: serial block face; FIB- and SBF-SEM used fixed platelet samples, CT- and WP-TEM used native unfixed platelets; (*) RCF (Relative Centrifugal Force) estimated from the centrifuge parameters used; (**) platelets’ suspension was washed in presence of prostacyclin and apyrase and then fixed with glutaraldehyde. For the WP-TEM technique, the authors all refer to the publications of James G. White for the criteria for identifying dense-granules. For Chen and colleagues [67], it is mentioned that the dense-granules counts were performed within 48 h after sample collection by 1 of 4 electron microscopy (EM) expert users; for Brunet and colleagues [64], dense-granules were counted by 1 of 2 EM expert users; and, for Pennamem and colleagues [30], dense-granules were counted by only 1 EM expert user. A hypothesis that could explain the lowest normal counts observed by Chen and colleagues would be the selection of a subpopulation of platelets poor in granules because of a strong and long centrifugation. Another hypothesis would be the way of platelet-rich plasma (PRP) collection: for Pennamen and colleagues, PRP has been collected in full at the edge of the buffy coat layer to not exclude large platelets. The PRP preparation process is not mentioned in the other publications. In specialized laboratories of platelet function studies, it is usual to observe the PRP far above the buffy coat having only platelets in the sample. It is recommended for aggregation tests or Western blot preparations. However, for platelet electron microscopies, it is bettser to have a few leukocytes in a single preparation. Indeed, it can quickly help to diagnose platelet disease such as MYH9. When the sample is also used to prepare thin sections, we recommended the use of a part of the buffy coat to carry platelets in the case of severe thrombocytopenia.

## References

[1] Versteeg H.H., Heemskerk J.W., Levi M., Reitsma P.H. (2013). New fundamentals in hemostasis. Physiol. Rev..

[2] Sharda A., Flaumenhaft R. (2018). The life cycle of platelet granules. F1000Res.

[3] Gachet C. (2006). Regulation of platelet functions by P2 receptors. Annu. Rev. Pharmacol. Toxicol..

[4] Masliah-Planchon J., Darnige L., Bellucci S. (2013). Molecular determinants of platelet delta storage pool deficiencies: An update. Br. J. Haematol..

[5] Flaumenhaft R., Michelson A.D. (2013). Platelet Secretion—Chapter 18. Platelets.

[6] Starcevic M., Dell’Angelica E.C. (2004). Identification of snapin and three novel proteins (BLOS1, BLOS2, and BLOS3/reduced pigmentation) as subunits of biogenesis of lysosome-related organelles complex-1 (BLOC-1). J. Biol. Chem..

[7] Huang L., Kuo Y.M., Gitschier J. (1999). The pallid gene encodes a novel, syntaxin 13-interacting protein involved in platelet storage pool deficiency. Nat. Genet..

[8] Bultema J.J., Ambrosio A.L., Burek C.L., Di Pietro S.M. (2012). BLOC-2, AP-3, and AP-1 proteins function in concert with Rab38 and Rab32 proteins to mediate protein trafficking to lysosome-related organelles. J. Biol. Chem..

[9] Gerondopoulos A., Langemeyer L., Liang J.R., Linford A., Barr F.A. (2012). BLOC-3 mutated in Hermansky-Pudlak syndrome is a Rab32/38 guanine nucleotide exchange factor. Curr. Biol. CB.

[10] Jedlitschky G., Greinacher A., Kroemer H.K. (2012). Transporters in human platelets: Physiologic function and impact for pharmacotherapy. Blood.

[11] Ambrosio A.L., Boyle J.A., Di Pietro S.M. (2012). Mechanism of platelet dense granule biogenesis: Study of cargo transport and function of Rab32 and Rab38 in a model system. Blood.

[12] Peden A.A., Oorschot V., Hesser B.A., Austin C.D., Scheller R.H., Klumperman J. (2004). Localization of the AP-3 adaptor complex defines a novel endosomal exit site for lysosomal membrane proteins. J. Cell Biol..

[13] Di Pietro S.M., Falcon-Perez J.M., Tenza D., Setty S.R., Marks M.S., Raposo G., Dell’Angelica E.C. (2006). BLOC-1 interacts with BLOC-2 and the AP-3 complex to facilitate protein trafficking on endosomes. Mol. Biol. Cell.

[14] Carneiro A.M., Cook E.H., Murphy D.L., Blakely R.D. (2008). Interactions between integrin alphaIIbbeta3 and the serotonin transporter regulate serotonin transport and platelet aggregation in mice and humans. J. Clin. Investig..

[15] Decouture B., Dreano E., Belleville-Rolland T., Kuci O., Dizier B., Bazaa A., Coqueran B., Lompre A.M., Denis C.V., Hulot J.S. (2015). Impaired platelet activation and cAMP homeostasis in MRP4-deficient mice. Blood.

[16] Holmsen H., Weiss H.J. (1972). Further evidence for a deficient storage pool of adenine nucleotides in platelets from some patients with thrombocytopathia—“Storage pool disease”. Blood.

[17] Holmsen H., Dangelmaier C.A. (1989). Measurement of secretion of adenine nucleotides. Methods Enzym..

[18] Morrissey J.H. (2012). Polyphosphate: A link between platelets, coagulation and inflammation. Int. J. Hematol..

[19] Smith S.A., Morrissey J.H. (2008). Polyphosphate enhances fibrin clot structure. Blood.

[20] Golebiewska E.M., Harper M.T., Williams C.M., Savage J.S., Goggs R., Fischer von Mollard G., Poole A.W. (2015). Syntaxin 8 regulates platelet dense granule secretion, aggregation, and thrombus stability. J. Biol. Chem..

[21] Bolton-Maggs P.H., Chalmers E.A., Collins P.W., Harrison P., Kitchen S., Liesner R.J., Minford A., Mumford A.D., Parapia L.A., Perry D.J. (2006). A review of inherited platelet disorders with guidelines for their management on behalf of the UKHCDO. Br. J. Haematol..

[22] Barbosa M.D., Nguyen Q.A., Tchernev V.T., Ashley J.A., Detter J.C., Blaydes S.M., Brandt S.J., Chotai D., Hodgman C., Solari R.C. (1996). Identification of the homologous beige and Chediak-Higashi syndrome genes. Nature.

[23] Sepulveda F.E., Burgess A., Heiligenstein X., Goudin N., Menager M.M., Romao M., Cote M., Mahlaoui N., Fischer A., Raposo G. (2015). LYST controls the biogenesis of the endosomal compartment required for secretory lysosome function. Traffic.

[24] Jin Y., Zhang L., Wang S., Chen F., Gu Y., Hong E., Yu Y., Ni X., Guo Y., Shi T. (2017). Whole Genome Sequencing Identifies Novel Compound Heterozygous Lysosomal Trafficking Regulator Gene Mutations Associated with Autosomal Recessive Chediak-Higashi Syndrome. Sci. Rep..

[25] Martina J.A., Moriyama K., Bonifacino J.S. (2003). BLOC-3, a protein complex containing the Hermansky-Pudlak syndrome gene products HPS1 and HPS4. J. Biol. Chem..

[26] Feng L., Seymour A.B., Jiang S., To A., Peden A.A., Novak E.K., Zhen L., Rusiniak M.E., Eicher E.M., Robinson M.S. (1999). The beta3A subunit gene (Ap3b1) of the AP-3 adaptor complex is altered in the mouse hypopigmentation mutant pearl, a model for Hermansky-Pudlak syndrome and night blindness. Hum. Mol. Genet..

[27] Di Pietro S.M., Falcon-Perez J.M., Dell’Angelica E.C. (2004). Characterization of BLOC-2, a complex containing the Hermansky-Pudlak syndrome proteins HPS3, HPS5 and HPS6. Traffic.

[28] Li W., Zhang Q., Oiso N., Novak E.K., Gautam R., O’Brien E.P., Tinsley C.L., Blake D.J., Spritz R.A., Copeland N.G. (2003). Hermansky-Pudlak syndrome type 7 (HPS-7) results from mutant dysbindin, a member of the biogenesis of lysosome-related organelles complex 1 (BLOC-1). Nat. Genet..

[29] Morgan N.V., Pasha S., Johnson C.A., Ainsworth J.R., Eady R.A., Dawood B., McKeown C., Trembath R.C., Wilde J., Watson S.P. (2006). A germline mutation in BLOC1S3/reduced pigmentation causes a novel variant of Hermansky-Pudlak syndrome (HPS8). Am. J. Hum. Genet..

[30] Pennamen P., Le L., Tingaud-Sequeira A., Fiore M., Bauters A., Van Duong Beatrice N., Coste V., Bordet J.C., Plaisant C., Diallo M. (2020). BLOC1S5 pathogenic variants cause a new type of Hermansky-Pudlak syndrome. Genet. Med. Off. J. Am. Coll. Med. Genet..

[31] Cullinane A.R., Curry J.A., Carmona-Rivera C., Summers C.G., Ciccone C., Cardillo N.D., Dorward H., Hess R.A., White J.G., Adams D. (2011). A BLOC-1 mutation screen reveals that PLDN is mutated in Hermansky-Pudlak Syndrome type 9. Am. J. Hum. Genet..

[32] Ammann S., Schulz A., Krageloh-Mann I., Dieckmann N.M., Niethammer K., Fuchs S., Eckl K.M., Plank R., Werner R., Altmuller J. (2016). Mutations in AP3D1 associated with immunodeficiency and seizures define a new type of Hermansky-Pudlak syndrome. Blood.

[33] Mohammed M., Al-Hashmi N., Al-Rashdi S., Al-Sukaiti N., Al-Adawi K., Al-Riyami M., Al-Maawali A. (2019). Biallelic mutations in AP3D1 cause Hermansky-Pudlak syndrome type 10 associated with immunodeficiency and seizure disorder. Eur. J. Med. Genet..

[34] Huizing M., Malicdan M.C.V., Wang J.A., Pri-Chen H., Hess R.A., Fischer R., O’Brien K.J., Merideth M.A., Gahl W.A., Gochuico B.R. (2020). Hermansky-Pudlak syndrome: Mutation update. Hum. Mutat..

[35] Griscelli C., Durandy A., Guy-Grand D., Daguillard F., Herzog C., Prunieras M. (1978). A syndrome associating partial albinism and immunodeficiency. Am. J. Med..

[36] Westbroek W., Tuchman M., Tinloy B., De Wever O., Vilboux T., Hertz J.M., Hasle H., Heilmann C., Helip-Wooley A., Kleta R. (2008). A novel missense mutation (G43S) in the switch I region of Rab27A causing Griscelli syndrome. Mol. Genet. Metab..

[37] White J.G., Keel S., Reyes M., Burris S.M. (2007). Alpha-delta platelet storage pool deficiency in three generations. Platelets.

[38] Ferreira C.R., Chen D., Abraham S.M., Adams D.R., Simon K.L., Malicdan M.C., Markello T.C., Gunay-Aygun M., Gahl W.A. (2017). Combined alpha-delta platelet storage pool deficiency is associated with mutations in GFI1B. Mol. Genet. Metab..

[39] Jedlitschky G., Cattaneo M., Lubenow L.E., Rosskopf D., Lecchi A., Artoni A., Motta G., Niessen J., Kroemer H.K., Greinacher A. (2010). Role of MRP4 (ABCC4) in platelet adenine nucleotide-storage: Evidence from patients with delta-storage pool deficiencies. Am. J. Pathol..

[40] Mumford A.D., Frelinger A.L., Gachet C., Gresele P., Noris P., Harrison P., Mezzano D. (2015). A review of platelet secretion assays for the diagnosis of inherited platelet secretion disorders. Thromb. Haemost..

[41] Quiroga T., Goycoolea M., Panes O., Aranda E., Martinez C., Belmont S., Munoz B., Zuniga P., Pereira J., Mezzano D. (2007). High prevalence of bleeders of unknown cause among patients with inherited mucocutaneous bleeding. A prospective study of 280 patients and 299 controls. Haematologica.

[42] Mezzano D., Quiroga T., Pereira J. (2009). The level of laboratory testing required for diagnosis or exclusion of a platelet function disorder using platelet aggregation and secretion assays. Semin. Thromb. Hemost..

[43] Fiore M., Garcia C., Sié P., Favier R., Lavenu-Bombled C., Hurtaud M.F., Gachet C., Alessi M.C., Dupuis A. (2017). Déficit en granules denses plaquettaires: Une cause sous-estimée de saignements inexpliqués. Hématologie.

[44] Gresele P., Harrison P., Bury L., Falcinelli E., Gachet C., Hayward C.P., Kenny D., Mezzano D., Mumford A.D., Nugent D. (2014). Diagnosis of suspected inherited platelet function disorders: Results of a worldwide survey. J. Thromb. Haemost..

[45] Kerenyi A., Schlammadinger A., Ajzner E., Szegedi I., Kiss C., Pap Z., Boda Z., Muszbek L. (1999). Comparison of PFA-100 closure time and template bleeding time of patients with inherited disorders causing defective platelet function. Thromb. Res..

[46] Hechler B., Dupuis A., Mangin P.H., Gachet C. (2019). Platelet preparation for function testing in the laboratory and clinic: Historical and practical aspects. Res. Pract. Thromb. Haemost..

[47] Nieuwenhuis H.K., Akkerman J.W., Sixma J.J. (1987). Patients with a prolonged bleeding time and normal aggregation tests may have storage pool deficiency: Studies on one hundred six patients. Blood.

[48] Ingerman C.M., Smith J.B., Shapiro S., Sedar A., Silver M.J. (1978). Hereditary abnormality of platelet aggregation attributable to nucleotide storage pool deficiency. Blood.

[49] Cattaneo M., Cerletti C., Harrison P., Hayward C.P., Kenny D., Nugent D., Nurden P., Rao A.K., Schmaier A.H., Watson S.P. (2013). Recommendations for the Standardization of Light Transmission Aggregometry: A Consensus of the Working Party from the Platelet Physiology Subcommittee of SSC/ISTH. J. Thromb. Haemost..

[50] Lanza F., Beretz A., Stierle A., Hanau D., Kubina M., Cazenave J.P. (1988). Epinephrine potentiates human platelet activation but is not an aggregating agent. Am. J. Physiol..

[51] Kinlough-Rathbone R.L., Mustard J.F., Packham M.A., Perry D.W., Reimers H.J., Cazenave J.P. (1977). Properties of washed human platelets. Thromb. Haemost..

[52] Cazenave J.P., Ohlmann P., Cassel D., Eckly A., Hechler B., Gachet C. (2004). Preparation of washed platelet suspensions from human and rodent blood. Methods Mol. Biol..

[53] Bull B.S. (1966). The ultrastructure of negatively stained platelets. Some physiologic implications. Blood.

[54] White J.G. (1969). The dense bodies of human platelets: Inherent electron opacity of the serotonin storage particles. Blood.

[55] Martin J.H., Carson F.L., Race G.J. (1974). Calcium-containing platelet granules. J. Cell Biol..

[56] Skaer R.J., Peters P.D., Emmines J.P. (1974). The localization of calcium and phosphorus in human platelets. J. Cell Sci..

[57] Born G.V., Ingram G.I., Stacey R.S. (1958). The relationship between 5-hydroxytryptamine and adenosine triphosphate in blood platelets. Br. J. Pharmacol. Chemother..

[58] Fukami M.H., Dangelmaier C.A., Bauer J.S., Holmsen H. (1980). Secretion, subcellular localization and metabolic status of inorganic pyrophosphate in human platelets. A major constituent of the amine-storing granules. Biochem. J..

[59] Ruiz F.A., Lea C.R., Oldfield E., Docampo R. (2004). Human platelet dense granules contain polyphosphate and are similar to acidocalcisomes of bacteria and unicellular eukaryotes. J. Biol. Chem..

[60] Donovan A.J., Kalkowski J., Smith S.A., Morrissey J.H., Liu Y. (2014). Size-controlled synthesis of granular polyphosphate nanoparticles at physiologic salt concentrations for blood clotting. Biomacromolecules.

[61] Kornberg A. (1995). Inorganic polyphosphate: Toward making a forgotten polymer unforgettable. J. Bacteriol..

[62] Mailer R.K.W., Hanel L., Allende M., Renne T. (2019). Polyphosphate as a Target for Interference With Inflammation and Thrombosis. Front. Med. (Lausanne).

[63] Baker C.J., Smith S.A., Morrissey J.H. (2019). Polyphosphate in thrombosis, hemostasis, and inflammation. Res. Pract. Thromb. Haemost..

[64] Verhoef J.J., Barendrecht A.D., Nickel K.F., Dijkxhoorn K., Kenne E., Labberton L., McCarty O.J., Schiffelers R., Heijnen H.F., Hendrickx A.P. (2017). Polyphosphate nanoparticles on the platelet surface trigger contact system activation. Blood.

[65] White J.G. (2008). Electron opaque structures in human platelets: Which are or are not dense bodies?. Platelets.

[66] Brunet J.G., Iyer J.K., Badin M.S., Graf L., Moffat K.A., Timleck M., Spitzer E., Hayward C.P.M. (2018). Electron microscopy examination of platelet whole mount preparations to quantitate platelet dense granule numbers: Implications for diagnosing suspected platelet function disorders due to dense granule deficiency. Int. J. Lab. Hematol..

[67] Chen D., Uhl C.B., Bryant S.C., Krumwiede M., Barness R.L., Olson M.C., Gossman S.C., Erdogan Damgard S., Gamb S.I., Cummins L.A. (2018). Diagnostic laboratory standardization and validation of platelet transmission electron microscopy. Platelets.

[68] Weiss H.J., Lages B., Vicic W., Tsung L.Y., White J.G. (1993). Heterogeneous abnormalities of platelet dense granule ultrastructure in 20 patients with congenital storage pool deficiency. Br. J. Haematol..

[69] Gunning W.T., Raghavan M., Calomeni E.P., Turner J.N., Roysam B., Roysam S., Smith M.R., Kouides P.A., Lachant N.A. (2020). A Morphometric Analysis of Platelet Dense Granules of Patients with Unexplained Bleeding: A New Entity of Delta-Microgranular Storage Pool Deficiency. J. Clin. Med..

[70] Markello T., Chen D., Kwan J.Y., Horkayne-Szakaly I., Morrison A., Simakova O., Maric I., Lozier J., Cullinane A.R., Kilo T. (2015). York platelet syndrome is a CRAC channelopathy due to gain-of-function mutations in STIM1. Mol. Genet. Metab..

[71] Gunning W.T., Calomeni E.P. (2000). A brief review of transmission electron microscopy and applications in pathology. J. Histotechnol..

[72] Sorokin V., Alkhoury R., Al-Rawabdeh S., Houston R.H., Thornton D., Kerlin B., O’Brien S., Baker P., Boesel C., Uddin M. (2016). Reference Range of Platelet Delta Granules in the Pediatric Age Group: An Ultrastructural Study of Platelet Whole Mount Preparations from Healthy Volunteers. Pediatr. Dev. Pathol..

[73] Wang R., Stone R.L., Kaelber J.T., Rochat R.H., Nick A.M., Vijayan K.V., Afshar-Kharghan V., Schmid M.F., Dong J.F., Sood A.K. (2015). Electron cryotomography reveals ultrastructure alterations in platelets from patients with ovarian cancer. Proc. Natl. Acad. Sci. USA.

[74] Eckly A., Rinckel J.Y., Proamer F., Ulas N., Joshi S., Whiteheart S.W., Gachet C. (2016). Respective contributions of single and compound granule fusion to secretion by activated platelets. Blood.

[75] Pokrovskaya I.D., Yadav S., Rao A., McBride E., Kamykowski J.A., Zhang G., Aronova M.A., Leapman R.D., Storrie B. (2020). 3D ultrastructural analysis of alpha-granule, dense granule, mitochondria, and canalicular system arrangement in resting human platelets. Res. Pract. Thromb. Haemost..

[76] Saultier P., Vidal L., Canault M., Bernot D., Falaise C., Pouymayou C., Bordet J.C., Saut N., Rostan A., Baccini V. (2017). Macrothrombocytopenia and dense granule deficiency associated with FLI1 variants: Ultrastructural and pathogenic features. Haematologica.

[77] Asher L., Hata J. (2020). Platelet Electron Microscopy: Utilizing LEAN Methodology to Optimize Laboratory Workflow. Pediatr. Dev. Pathol..

[78] Marneth A.E., van Heerde W.L., Hebeda K.M., Laros-van Gorkom B.A., Barteling W., Willemsen B., de Graaf A.O., Simons A., Jansen J.H., Preijers F. (2017). Platelet CD34 expression and alpha/delta-granule abnormalities in GFI1B- and RUNX1-related familial bleeding disorders. Blood.

[79] Urban D., Pluthero F.G., Christensen H., Baidya S., Rand M.L., Das A., Shah P.S., Chitayat D., Blanchette V.S., Kahr W.H. (2017). Decreased numbers of dense granules in fetal and neonatal platelets. Haematologica.

[80] Nessle C.N., Ghosal S., Mathews C., Taylor D., Myers J., Raj A., Panigrahi A. (2019). Weak correlation of bleeding scores to platelet electron microscopy: A retrospective chart review of pediatric patients with delta-storage pool disorder. Pediatr. Blood Cancer.

[81] Guicheney P. (1988). Human platelet serotonin content: Methodological aspects and physiological variations. Methods Find. Exp. Clin. Pharmacol..

[82] Flachaire E., Beney C., Berthier A., Salandre J., Quincy C., Renaud B. (1990). Determination of reference values for serotonin concentration in platelets of healthy newborns, children, adults, and elderly subjects by HPLC with electrochemical detection. Clin. Chem..

[83] Gerrard J.M., Rao G.H., White J.G. (1977). The influence of reserpine and ethylenediaminetetraacetic acid (EDTA) on serotonin storage organelles of blood platelets. Am. J. Pathol..

[84] Maurer-Spurej E., Pittendreigh C., Solomons K. (2004). The influence of selective serotonin reuptake inhibitors on human platelet serotonin. Thromb. Haemost..

[85] Skaer R.J., Flemans R.J., McQuilkan S. (1981). Mepacrine stains the dense bodies of human platelets and not platelet lysosomes. Br. J. Haematol..

[86] Greinacher A., Pecci A., Kunishima S., Althaus K., Nurden P., Balduini C.L., Bakchoul T. (2017). Diagnosis of inherited platelet disorders on a blood smear: A tool to facilitate worldwide diagnosis of platelet disorders. J. Thromb. Haemost..

[87] Zaninetti C., Greinacher A. (2020). Diagnosis of Inherited Platelet Disorders on a Blood Smear. J. Clin. Med..

[88] Westmoreland D., Shaw M., Grimes W., Metcalf D.J., Burden J.J., Gomez K., Knight A.E., Cutler D.F. (2016). Super-resolution microscopy as a potential approach to diagnosis of platelet granule disorders. J. Thromb. Haemost..

[89] Holmsen H., Ostvold A.C., Day H.J. (1973). Behaviour of endogenous and newly absorbed serotonin in the platelet release reaction. Biochem. Pharmacol..

[90] Anderson G.M., Hall L.M., Yang J.X., Cohen D.J. (1992). Platelet dense granule release reaction monitored by high-performance liquid chromatography-fluorometric determination of endogenous serotonin. Anal. Biochem..

[91] Bossant M.J., Ninio E., Delautier D., Bessou G., Trouvin J.H., Benveniste J. (1989). Quantitation of paf-acether by release of endogenous platelet serotonin assessed by liquid chromatography with electrochemical detection. Anal. Biochem..

[92] Ge S., Wittenberg N.J., Haynes C.L. (2008). Quantitative and real-time detection of secretion of chemical messengers from individual platelets. Biochemistry.

[93] Ge S., Woo E., White J.G., Haynes C.L. (2011). Electrochemical measurement of endogenous serotonin release from human blood platelets. Anal. Chem..

[94] Gawaz M.P., Bogner C., Gurland H.J. (1993). Flow-cytometric analysis of mepacrine-labelled platelets in patients with end-stage renal failure. Haemostasis.

[95] van Asten I., Blaauwgeers M., Granneman L., Heijnen H.F.G., Kruip M., Beckers E.A.M., Coppens M., Eikenboom J., Tamminga R.Y.J., Pasterkamp G. (2020). Flow cytometric mepacrine fluorescence can be used for the exclusion of platelet dense granule deficiency. J. Thromb. Haemost..

[96] Aschar-Sobbi R., Abramov A.Y., Diao C., Kargacin M.E., Kargacin G.J., French R.J., Pavlov E. (2008). High sensitivity, quantitative measurements of polyphosphate using a new DAPI-based approach. J. Fluoresc..

[97] Schlagenhauf A., Pohl S., Haidl H., Leschnik B., Gallistl S., Muntean W. (2016). Non-enzymatic quantification of polyphosphate levels in platelet lysates and releasates. J. Pharm. Biomed. Anal..

[98] Smith S.A., Morrissey J.H. (2007). Sensitive fluorescence detection of polyphosphate in polyacrylamide gels using 4′, 6-diamidino-2-phenylindol. Electrophoresis.

[99] Feinman R.D., Lubowsky J., Charo I., Zabinski M.P. (1977). The lumi-aggregometer: A new instrument for simultaneous measurement of secretion and aggregation by platelets. J. Lab. Clin. Med..

[100] Cattaneo M. (2009). Light transmission aggregometry and ATP release for the diagnostic assessment of platelet function. Semin. Thromb. Hemost..

[101] Badin M.S., Graf L., Iyer J.K., Moffat K.A., Seecharan J.L., Hayward C.P. (2016). Variability in platelet dense granule adenosine triphosphate release findings amongst patients tested multiple times as part of an assessment for a bleeding disorder. Int. J. Lab. Hematol..

[102] Stockley J., Morgan N.V., Bem D., Lowe G.C., Lordkipanidze M., Dawood B., Simpson M.A., Macfarlane K., Horner K., Leo V.C. (2013). Enrichment of FLI1 and RUNX1 mutations in families with excessive bleeding and platelet dense granule secretion defects. Blood.

[103] Latger-Cannard V., Philippe C., Bouquet A., Baccini V., Alessi M.C., Ankri A., Bauters A., Bayart S., Cornillet-Lefebvre P., Daliphard S. (2016). Haematological spectrum and genotype-phenotype correlations in nine unrelated families with RUNX1 mutations from the French network on inherited platelet disorders. Orphanet J. Rare Dis..

[104] Ringeisen A.L., Schimmenti L.A., White J.G., Schoonveld C., Summers C.G. (2013). Hermansky-Pudlak syndrome (HPS5) in a nonagenarian. J. AAPOS.

[105] Novak E.K., Hui S.W., Swank R.T. (1984). Platelet storage pool deficiency in mouse pigment mutations associated with seven distinct genetic loci. Blood.

[106] Cai H., Mullier F., Frotscher B., Briquel M.E., Toussaint M., Massin F., Lecompte T., Latger-Cannard V. (2016). Usefulness of Flow Cytometric Mepacrine Uptake/Release Combined with CD63 Assay in Diagnosis of Patients with Suspected Platelet Dense Granule Disorder. Semin. Thromb. Hemost..

[107] Rodeghiero F., Tosetto A., Abshire T., Arnold D.M., Coller B., James P., Neunert C., Lillicrap D. (2010). ISTH/SSC bleeding assessment tool: A standardized questionnaire and a proposal for a new bleeding score for inherited bleeding disorders. J. Thromb. Haemost..

[108] Selle F., James C., Tuffigo M., Pillois X., Viallard J.F., Alessi M.C., Fiore M. (2017). Clinical and Laboratory Findings in Patients with delta-Storage Pool Disease: A Case Series. Semin. Thromb. Hemost..

[109] Orsini S., Noris P., Bury L., Heller P.G., Santoro C., Kadir R.A., Butta N.C., Falcinelli E., Cid A.R., Fabris F. (2017). Bleeding risk of surgery and its prevention in patients with inherited platelet disorders. Haematologica.

[110] Civaschi E., Klersy C., Melazzini F., Pujol-Moix N., Santoro C., Cattaneo M., Lavenu-Bombled C., Bury L., Minuz P., Nurden P. (2015). Analysis of 65 pregnancies in 34 women with five different forms of inherited platelet function disorders. Br. J. Haematol..

[111] Dupuis A., Gachet C. (2018). Inherited platelet disorders: Management of the bleeding risk. Transfus. Clin. Biol. J. Soc. Fr. Transfus. Sang..

